# Peptidoglycan in osteoarthritis synovial tissue is associated with joint inflammation

**DOI:** 10.1186/s13075-024-03293-x

**Published:** 2024-03-27

**Authors:** Meaghan N Holub, Amanda Wahhab, Joseph R Rouse, Rebecca Danner, Lauren G Hackner, Christine B Duris, Mecaila E McClune, Jules M Dressler, Klemen Strle, Brandon L Jutras, Adam I Edelstein, Robert B Lochhead

**Affiliations:** 1 Department of Orthopaedic Surgery, Medical College of Wisconsin, BSB room 2850, Milwaukee, WI 53226 USA; 2 Department of Microbiology and Immunology, Medical College of Wisconsin, Milwaukee, WI USA; 3 Department of Pathology, Children’s Hospital of Wisconsin and the Medical College of Wisconsin, Milwaukee, WI USA; 4 Department of Biochemistry, Virginia Tech, Blacksburg, VA USA; 5 Fralin Life Sciences Institute, Virginia Tech, Blacksburg, VA USA; 6 Center for Emerging, Zoonotic, and Arthropod-borne Pathogens, Virginia Tech, Blacksburg, VA USA; 7 Department of Molecular Biology and Microbiology, Tufts University, Boston, MA USA; 8 Department of Orthopaedic Surgery, Northwestern University Feinberg School of Medicine, Chicago, IL USA; 9 Division of Rheumatology, Department of Medicine, Medical College of Wisconsin, Milwaukee, WI USA

**Keywords:** Osteoarthritis, Peptidoglycan, Synovitis, Inflammation

## Abstract

**Objectives:**

Peptidoglycan (PG) is an arthritogenic bacterial cell wall component whose role in human osteoarthritis is poorly understood. The purpose of this study was to determine if PG is present in synovial tissue of osteoarthritis patients at the time of primary total knee arthroplasty (TKA), and if its presence is associated with inflammation and patient reported outcomes.

**Methods:**

Intraoperative synovial tissue and synovial fluid samples were obtained from 56 patients undergoing primary TKA, none of whom had history of infection. PG in synovial tissue was detected by immunohistochemistry (IHC) and immunofluorescence microscopy (IFM). Synovial tissue inflammation and fibrosis were assessed by histopathology and synovial fluid cytokine quantification. Primary human fibroblasts isolated from arthritis synovial tissue were stimulated with PG to determine inflammatory cytokine response.

**Results:**

A total of 33/56 (59%) of primary TKA synovial tissue samples were positive for PG by IHC, and PG staining colocalized with markers of synovial macrophages and fibroblasts by IFM. Synovial tissue inflammation and elevated IL-6 in synovial fluid positively correlated with PG positivity. Primary human fibroblasts stimulated with PG secreted high levels of IL-6, consistent with ex vivo findings. Interestingly, we observed a significant inverse correlation between PG and age at time of TKA, indicating younger age at time of TKA was associated with higher PG levels.

**Conclusion:**

Peptidoglycan is commonly found in synovial tissue from patients undergoing TKA. Our data indicate that PG may play an important role in inflammatory synovitis, particularly in patients who undergo TKA at a relatively younger age.

**Supplementary Information:**

The online version contains supplementary material available at 10.1186/s13075-024-03293-x.

## Introduction

Osteoarthritis (OA) of the knee affects over one-third of the United States population aged 60 years or greater [1]. The incidence of knee OA is expected to increase over the coming decades owing to the aging population and increases in obesity [2]. Pain and functional limitations from knee OA have major impacts on quality of life for persons living with arthritis [3], and surgical management of lower extremity arthritis now comprises the largest procedural expenditure in the Medicare budget [4]. Knee replacement surgery is associated with improvements in pain and function [5], but 15–20% of patients have continued pain and dissatisfaction following surgery [6, 7].

Osteoarthritis is characterized macroscopically by loss of articular cartilage and changes to subchondral bone, involving changes to all tissues in the joint through a complex interplay of inflammatory molecules [8, 9]. Synovial inflammation and hypertrophy occur in joints affected by OA [10, 11], and severity of synovitis positively correlates with symptoms [12, 13]. Synovial inflammation present in OA is mediated by inflammatory cytokines [14], but disease mechanisms of inflammatory OA are incompletely understood. Owing to the growing disease burden of knee OA and the cost and imperfect outcomes associated with its treatment, there remains a critical need to better understand the pathophysiology of synovial inflammation both before and after knee replacement.

Microbial products derived from the microbiome have been proposed to contribute to joint inflammation in OA [15], and there is increasing evidence that circulating microbial debris contributes to OA [16]. Peptidoglycan (PG), a structural component of the bacterial cell wall and a highly conserved pathogen-associated molecular pattern (PAMP), has been shown to trigger inflammatory responses in both Lyme arthritis (LA) and rheumatoid arthritis (RA) [17–19]. PG is recognized by innate immune cells via pattern recognition receptors [18, 20, 21]. Bacterial DNA and bacterial debris, including PG, have also been reported in synovium of limited cohorts of patients with OA [22–24], but the prevalence and potential impact of PG in synovium in patients with advanced knee arthritis remain incompletely understood.

The aim of this study was to characterize the prevalence of PG in synovial fluid and tissue samples at time of total knee arthroplasty performed for advanced OA, and to define its association with synovitis, inflammatory cytokines, and patient outcomes.

## Methods

### Patients

The present study was approved by the Medical College of Wisconsin and Froedtert Hospital Institutional Review Board (IRB) for Human Subject Research (PRO00035381, “Arthritis research at MCW”). Written informed consent was obtained from 66 patients undergoing elective, primary TKA with one of the senior authors (AE). None of the patients had a prior history of knee infection, prior knee surgery, or had an intra-articular injection within three months of surgery. We also enrolled four patients undergoing debridement and component explant due to periprosthetic joint infections to serve as positive controls and to optimize immunostaining. Ten of the OA patient samples were excluded due to excessive, nonspecific background staining. Of the remaining 56 quality samples, 53 patients had been diagnosed with degenerative arthritis.

### Clinical evaluation

Patient demographics and comorbidities were collected during the pre-operative appointment. Knee injury and Osteoarthritis Outcome Score for Joint Replacement (KOOS JR) and Veterans Rand-12 Health Survey (VR-12) scores were collected at baseline and again at 3, 6, and 12 months postoperatively. All patients were followed clinically for at least one-year post-operatively to monitor recovery and occurrences of complications. Pain and functional recovery were assessed by patient reported outcome measures; occurrence of any infectious complications or reoperations were recorded.

### Specimen collection

At the beginning of each patient’s TKA procedure, synovial fluid was aspirated from the operative knee using an 18-gauge needle following sterile prepping and draping and skin incision but prior to arthrotomy. Following arthrotomy, synovial tissue was harvested from the suprapatellar pouch and the medial and lateral gutters. Specimen was stored in sterile specimen containers and prepared for various analyses within 6 h of collection.

### Specimen preparation and isolation

All synovial fluid and synovial tissue samples were collected and processed under sterile conditions and stored in -80 °C freezer or liquid nitrogen until further use. If available, synovial fluid was flash-frozen and stored for downstream cytokine analysis. Synovium was isolated from collected synovial tissue using sterile surgical scissors and forceps and sectioned into small (1–2 mm^3^) tissue fragments. Two sections from each patient were embedded within optimal cutting temperature compound. Samples were stored in a -80 °C freezer overnight, then transferred to liquid nitrogen for long term storage prior to histopathologic analysis.

### Histopathology

Two sections from each patient were used to assess inflammation by hematoxylin and eosin (H&E) stain and fibrosis by Masson’s trichrome stain. H&E-stained sections were qualitatively evaluated and blindly scored for markers of inflammatory synovitis on a scale of 0 to 3, with 3 being most severe. Three separate scores for overall inflammatory infiltrate, number of inflammatory foci, and synovial lining thickness were summed together to produce an overall inflammatory synovitis score for each patient sample. Each trichrome-stained section was scored in a blinded fashion using a scale of 0 to 3, with 3 being most severely fibrotic. All scores were independently reviewed prior to unblinding of the coded samples.

### Anti-peptidoglycan antibody generation

*B. burgdorferi* B31-A3 was cultured in complete BSK-II media supplemented with 6% rabbit serum. *Escherichia coli* strain K12; *Bacillus subtilis* strain 168; and *Staphylococcus aureus* (FDA 209); were propagated in Lysogeny Broth (LB), *Streptococcus mutans* strain Clark in Brain Heart Infusion (BHI) broth, and *Deinococcous radiodurans* strain 13,939 in Tryptone Yeast (TY) media supplemented with 10% glucose.

All bacteria were grown to mid-exponential phase, harvested at 4,000 x g for 15 min, and then washed twice with PBS. For peptidoglycan purification, bacterial pellets were resuspended in PBS and added dropwise into boiling SDS (5% w/v, final concentration) and boiled for 1 h as previously described [17]. All Gram-positive bacteria were bead-beat (BeadBug, Benchmark Scientific) prior to SDS boiling for 3 cycles of 60 s on, 60 s on ice. After boiling, all samples were cooled to 30ºC, and the pellets washed with autoclaved H_2_O four times using ultracentrifugation at 283,346 x g for 60 min at 30ºC. The pellets were then resuspended in H_2_O and treated with lipase (1 mg/ml) for 3 h, benzonase nuclease (4 µl/ml) for 2 h, and overnight with chymotrypsin (0.3 mg/ml), all with shaking at 37ºC. The next day 0.5% SDS was added to each pellet and heated to 80ºC for 30 min. The pellets were washed 3 times with autoclaved H_2_O at the same centrifugation conditions listed above. The Gram-positive samples were treated with a final concentration of 1 M HCl while continuously rotating at 4ºC for 48 h and centrifuged/washed 3 times, as described above. The dry weight was measured to quantify the amount of PG purified. To create the anti-peptidoglycan antibody, 5 BALB/cJ mice purchased from Jackson Laboratories were injected subcutaneously with 200 µg total of peptidoglycan from the bacteria listed above and mixed with equal parts of Freund’s Complete adjuvant (Thermo Scientific Ref: 77,140) (2 mg/ml final of PG). After 3 weeks all mice received a 265 µg booster injection of the same PG mixture. The mice were euthanized 2.5 weeks later and blood was collected. The blood was incubated at room temperature for 30 min prior to spinning at 1,500 x g for 10 min at 4ºC. The serum was then removed, pooled together, and frozen at -20ºC. The specificity of the antibody was tested using immunofluorescence and was found to bind *S. mutans, D. radiodurans*, *S. aureus*, and *E. coli* PG (data not shown) using methods described elsewhere [25, 26].

### PG staining and scoring

Two sections of tissue from each patient were coded and stained by immunohistochemistry using the mouse anti-PG antiserum to label PG in synovial tissue, followed by incubation with horseradish peroxidase-conjugated goat anti-mouse IgG (Sigma-Aldrich) as detection antibody. Non-immunized mouse serum was used as a negative control. Following staining optimization for the custom anti-serum, all sections were processed at one time by staff at our core facility to control for technical variability. Slides that had nonspecific edge staining artifacts were excluded from further analysis. For each Sect. (2 per patient), five 1mm^2^ fields were randomly selected from the tissue section and number of stained foci, corresponding to individual PG occurrences, were counted and summed across both Sect. (10 mm^2^ total area analyzed per patient sample). Samples were then scored from 0 to 4 based on the number of PG occurrences in tissue: 0 = no PG occurrences; 1 = 1–9 PG occurrences; 2 = 10–19 PG occurrences; 3 = 20–29 PG occurrences; 4 = 30 + PG occurrences.

### Immunofluorescence microscopy

Sections of synovial tissue were stained with mouse anti-PG antiserum (custom), goat anti-human CD90/Thy1 antibody (TA318808, Origene), rabbit anti-human CD68 (76,437, Cell Signaling) and DAPI (Sigma). Samples were then incubated with secondary (detection) antibodies: AF488-conjugated anti-mouse IgG (A10037, Invitrogen), Cy3-conjugated anti-rabbit IgG (715-166-152, Jackson Immuno), and AF647-conjugated anti-goat IgG (A21447, Invitrogen). Serial sections from the same patient stained with secondary antibodies only were performed for each sample to control for nonspecific staining. Slides were imaged using an Olympus VS120 slide scanner and images were analyzed using (LAUREN H ADD INFO HERE). Samples with high nonspecific staining of negative controls (primary antibodies omitted) were excluded from analysis.

### Primary human fibroblast isolation and stimulation

Fibroblasts were isolated from the human synovial tissue samples described above. A portion of the tissue fragments were transferred to a 15 ml conical centrifuge tube containing 5 ml of collagenase D (Sigma Aldrich 11,088,858,001) at a concentration of 1 mg/ml (dissolved in Hank’s balanced salt solution (HBSS) [Sigma Aldrich 55,037 C] + 1% Penicillin/Streptomycin (Pen/Strep) [Fisher Scientific 15,140,122]). The tube was kept in a 37 °C bead bath for 1 h and was shaken vigorously every 5 min to release cells. Large tissue fragments were removed using sterile forceps and discarded. Remaining liquid was centrifuged at 1100 rpm for 10 min at room temperature. Supernatant was discarded and cell pellet was resuspended in 5 ml of enriched human fibroblast medium (High glucose DMEM [Sigma Aldrich D5671] + 20% fetal bovine serum (FBS) [BioWest S1690] + 1% Pen/Strep + 1% glutamine [Fisher Scientific 35,050,061] + 1% non-essential amino acids (NEAA) [Fisher Scientific 11,140,050] + 5 ng/ml recombinant human FGF-basic [BioLegend 792,504]). Cells were then transferred to a T25 tissue culture flask and placed in a 37 °C incubator with 5% CO2. Cell culture medium was replaced every 3–4 days, and cells were passaged at ∼ 90% confluency. Primary fibroblasts were frozen at passage 4 and stored in liquid nitrogen.

### Fibroblast stimulation

Samples were passaged at least 6 times prior to use to enable isolation and expansion of fibroblasts. Cells were plated in 24-well plates at 2.5 × 10^5 cells per well in 500 µl of medium. Each patient sample was plated in two wells, and one of the wells was stimulated with 10 µg/ml of the muramyl dipeptide fragment from *Staphylococcus aureus* peptidoglycan (Sigma Aldrich 77,140) for 24 h. Cell culture supernatants were collected and stored at -80 °C until further analysis.

### Cytokine analysis

Cytokine analysis was performed using the LEGENDplex Human Inflammation Panel (Biolegend) to quantify 13 human inflammatory cytokines/chemokines (IL-1β, IFN-α2, IFN-γ, TNF-α, MCP-1 (CCL2), IL-6, IL-8 (CXCL8), IL-10, IL-12p70, IL-17 A, IL-18, IL-23, and IL-33). Bead populations conjugated with antibodies specific to the mentioned cytokines/chemokines were incubated with neat synovial fluid samples allowing the target analytes to bind to the specific capture bead. Biotinylated detection antibodies were then combined with the analyte bound beads and each detection antibody formed a bond with their specific analyte. Thereafter, Streptavidin-phycoerythrin (SA-PE) was added to bind to the biotinylated detection antibodies generating a fluorescent signal with an intensity proportionate to amount of the specific cytokine/chemokine bound to the capture bead. Each sample was run through a flow cytometer where SA-PE fluorescence intensity was converted to cytokine/chemokine concentration based on a standard concentration curve.

### Statistical analysis

Statistical associations between PG severity scores and synovial inflammation, accumulation of fibrotic tissue, cytokine levels, population demographics, and patient reported outcome scores were assessed using Pearson correlations and regression analysis (p value cutoff = 0.05). Statistically significant differences in cytokine secretion levels between stimulated vs. unstimulated fibroblasts were determined by paired two-tailed t test (p value cutoff = 0.05). All statistical analyses were performed using GraphPad Prism (v.9).

## Results

### Patient characteristics and outcomes

In total, 56 samples from patients undergoing primary, elective TKA met our staining quality and inclusion criteria. The average age and BMI of our patient cohort was 67 years and 31.5 kg/m^2^, respectively (Table 1). Post-operative patient outcomes were measured using KOOS JR and VR-12 scores (Table 1). As expected, there was significant improvement in scores from baseline to final follow up. There were no occurrences of periprosthetic joint infection throughout the follow up period for the elective TKA cohort, and no patients underwent revision surgery.

**Table 1:** Patient demographics and patient reported outcomes

	Baseline Characteristics	3 months	6 months	12 months
**Sex**	31/56 female (55%)			
**Age** median (SD)	67 (12.8)			
**BMI** median (SD)	31.5 (5.9)			
**White**	41 (73%)			
**Black**	10 (18%)			
**Asian**	3 (5%)			
**Other**	2 (4%)			
**KOOS JR** mean (SD)	40.3 (15.5)	59.5 (10.8)	69.2 (13.1)	79.5 (16.1)
**PCS-12** mean (SD)	30.7 (9.6)	38.7 (9.4)	46.3 [12]	49.5 (12.7)
**MCS-12** mean (SD)	46.2 (6.1)	44.1 (5.7)	43.3 (4.5)	43.1 (3.7)

Baseline characteristics and outcomes at follow up are shown. SD, standard deviation; KOOS JR, Knee Injury and Osteoarthritis Outcome Score for Joint Replacement; PCS-12, Physical Component Score for VR-12 outcome; MCS-12, Mental Component Score for VR-12 outcome.

### Identification of bacterial peptidoglycan within in synovial tissue

**Fig. 1:**
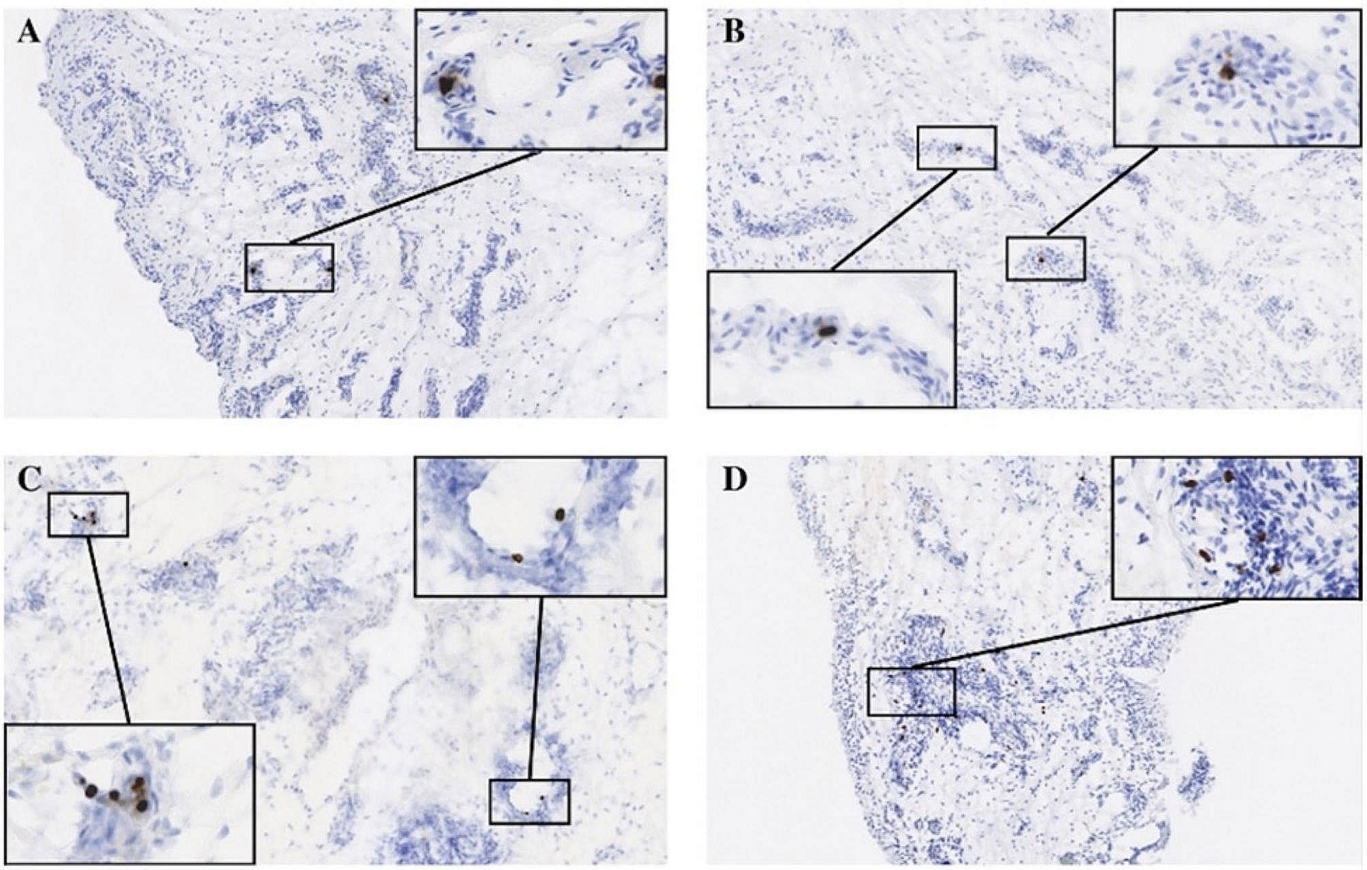
Detection of bacterial peptidoglycan (PG) in synovial tissue. Shown are representative sections of synovial tissue stained for PG by immunohistochemistry (see methods for details) obtained from (**A**) a patient with a periprosthetic joint infection (positive control), (**B**) a patient with a PG score of 2 (10–19 PG foci/10mm^2^), (**C**) a patient with a PG score of 3 (20–29 PG foci/10mm^2^), and (**D**) a patient with a PG score of 4 (30 + PG foci/10mm^2^). Enlarged insets show localization of PG foci (brown) within pockets of inflammation detectible by hematoxylin counterstain (blue)

To determine whether bacterial peptidoglycan was present in synovial tissue, we used immunohistochemistry (IHC) to stain for peptidoglycan (PG) in sections of fresh-frozen synovial tissue (Fig. 1). We validated our staining methodology using synovial tissue from 4 patients with periprosthetic joint infections to detect PG within the infected tissue (Fig. 1A). Using this validated method, we detected PG in 33/56 (59%) of synovial tissue from patients undergoing primary TKA with no history of joint infection. PG staining varied widely between patient samples, ranging from 0 to 94 PG occurrences per 10 mm^2^ of tissue.

**Fig. 2:**
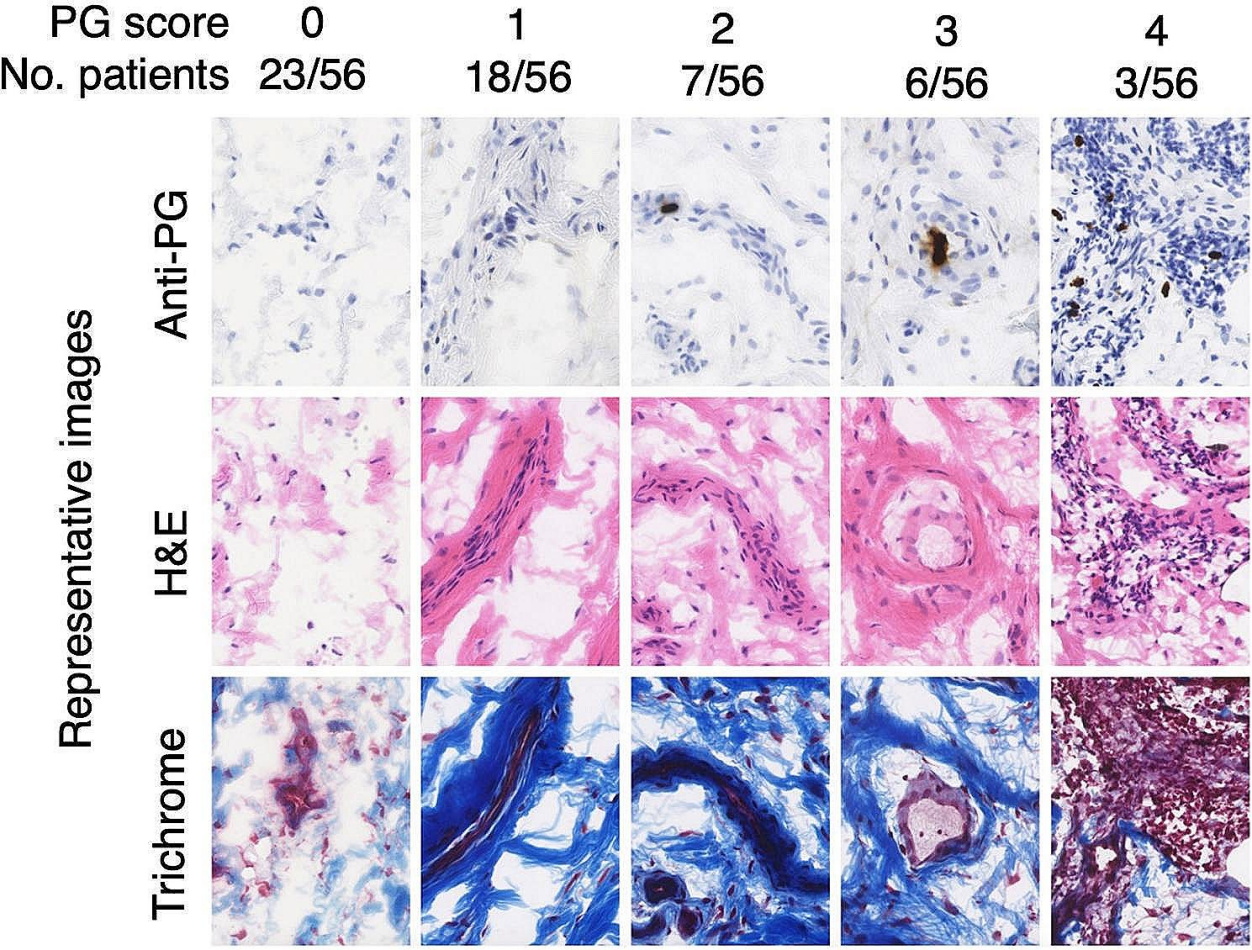
Association between peptidoglycan, synovial inflammation, and fibrosis. Shown are representative sections of synovial tissue stained for PG by immunohistochemistry (see methods for details). Inflammation and fibrosis were determined by H&E staining and Masson’s trichrome staining, respectively. Each column shows a representative sample that received a PG score 0–4 according to the quantity of PG staining foci per 10 mm^2^ (0 = none, 1 = 1–9, 2 = 10–19, 3 = 20–29, 4 = 30+). Inflammatory infiltrate and localized areas of fibrosis frequently colocalized with the regions of high PG staining intensity

Sample sections showed considerable variability in the degree of inflammation and fibrosis between patients, measured by H&E and trichrome staining, respectively (Fig. 2). PG in synovial tissue was typically localized within cells, and these PG-positive cells were often surrounded by foci of inflammatory infiltrate and/or regions of fibrosis.

### Correlations between synovial tissue PG and clinical and laboratory findings

**Fig. 3:**
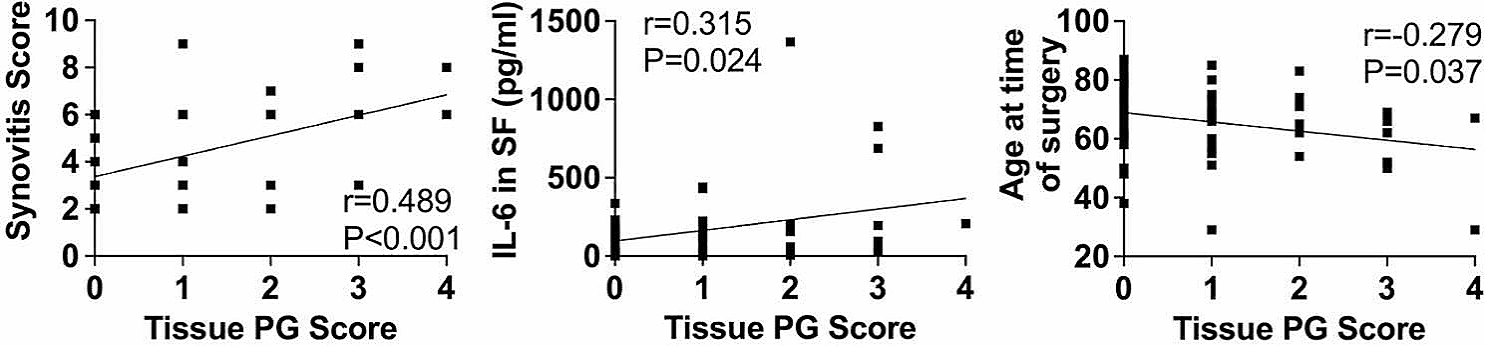
Correlations between synovial tissue PG score and clinical and laboratory findings. Pearson’s r values were calculated to determine correlations between tissue PG score (0–4) and clinical and laboratory data. Shown are the correlation curves for correlations between PG score and synovitis severity, IL-6 in synovial fluid (SF), and age at the time of surgery. Calculated Pearson’s r and P values are indicated in the figure

PG severity scores positively correlated with several clinical and laboratory findings (Fig. 3). Overall synovitis positively correlated with PG score (*r* = 0.489, *p* < 0.001). The level of IL-6 in synovial fluid also positively correlated with PG score (*r* = 0.315, *p* = 0.024). Additionally, there was a modest, significant inverse correlation between PG score and age at the time of surgery (*r*=-0.279, *p* = 0.037). Interestingly, there were no significant correlations between PG score and BMI, a well-studied risk factor for degenerative arthritis. Furthermore, there was no significant correlation between PG score and patient-reported outcomes.

### Cellular localization of PG-positive cells within synovial tissue

**Fig. 4:**
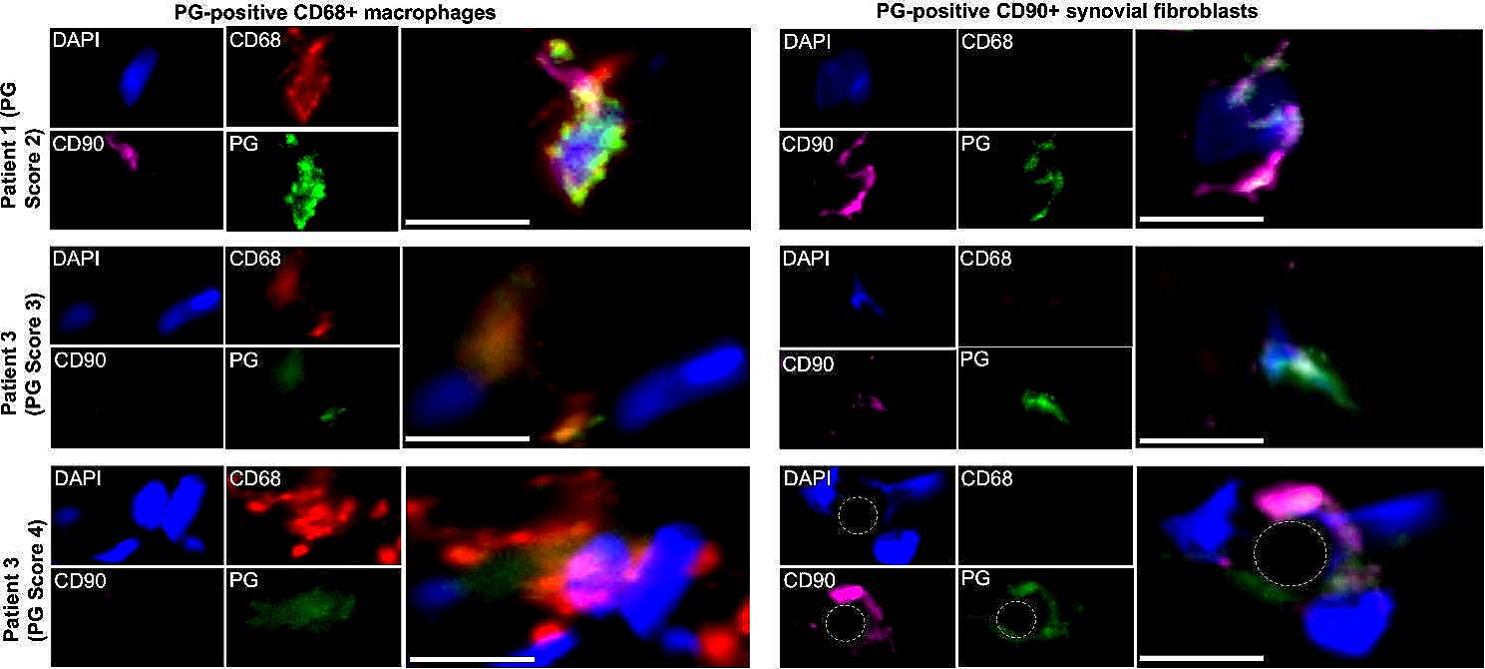
Colocalization of peptidoglycan with CD68 + macrophages and CD90 + synovial fibroblasts within synovial tissue. Shown are representative images of synovial tissue from three PG-positive patients (PG scores of 2, 3, and 4) stained for CD68 (macrophages, red), PG (green), and CD90 (synovial fibroblasts, magenta). DAPI was used as a nuclear stain. Images on left are representative of areas of PG-positive macrophages, and images on right are representative of areas of PG-positive synovial macrophages. Dotted circle indicates the approximate location of a small vessel within the inflamed synovium of Patient 3, and CD90 + cells in this image may be synovial fibroblasts, endothelial cells, or pericytes. Scale is indicated by a 10 μm white bar in each merged image

Staining for PG by IHC showed that cells of both mononuclear and fibroblastic morphology we positive for PG (Supplemental Fig. S1). We used immunofluorescence microscopy (IFM) to determine colocalization of PG with CD68 + macrophages and/or CD90 + synovial fibroblasts (Fig. 4). Three patients were selected representing PG scores of 2, 3, and 4. Most PG-positive cells colocalized with CD68 + macrophages. Some PG-positive CD90 + synovial fibroblasts were also observed in patients. CD90 expression in synovial tissue is not restricted to synovial fibroblasts, and some PG-positive CD90 + cells may be other stromal cells, such as endothelial cells or pericytes. However, due to technical challenges of using more than three markers for IFM imaging, further phenotypic resolution of PG-positive cells was not possible using this methodology.

### Inflammatory responses of synovial fibroblasts stimulated with PG

**Fig. 5:**
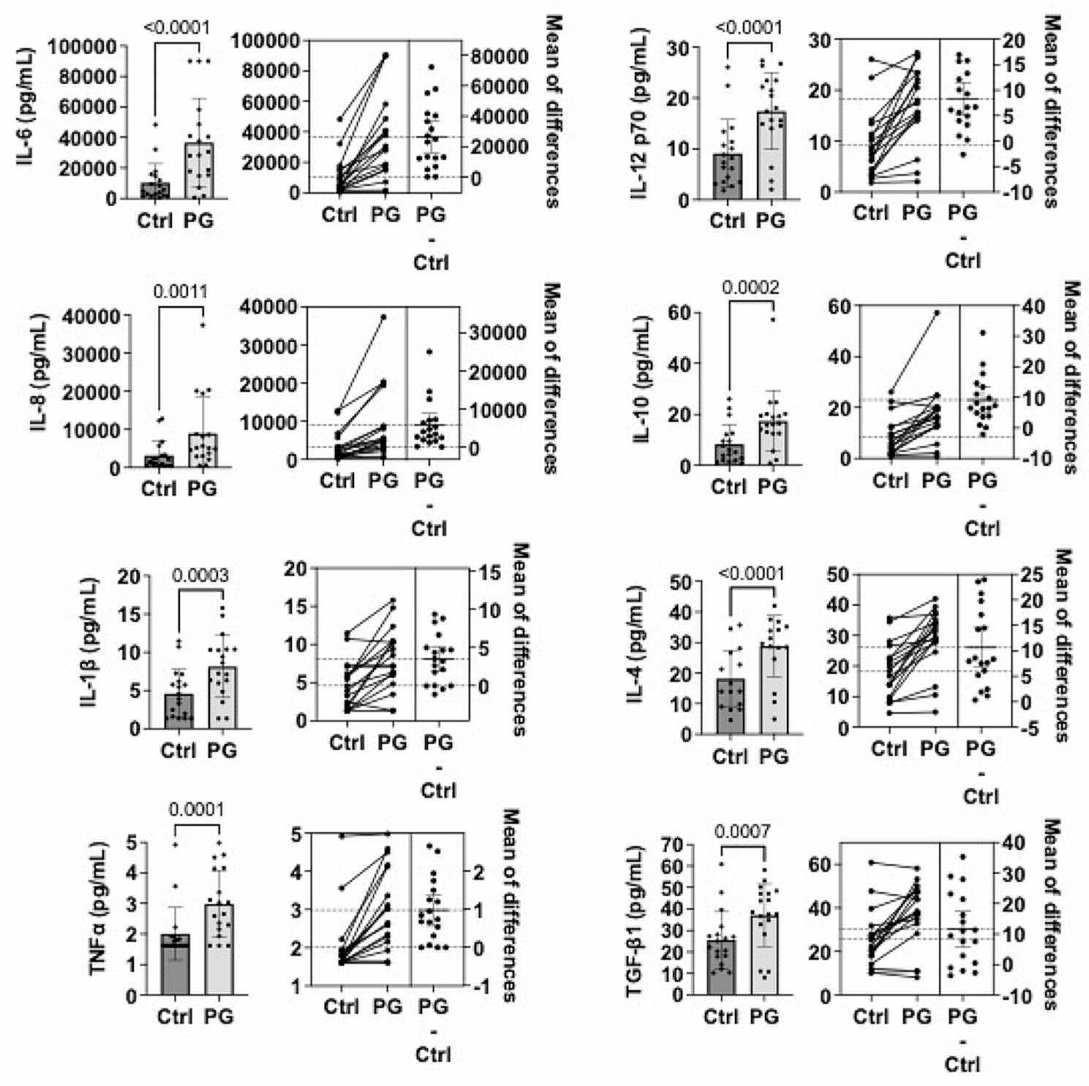
Cytokine secretion by primary human synovial fibroblasts stimulated with peptidoglycan (PG). Primary human synovial fibroblasts were isolated from 8 patients with osteoarthritis, 2 with joint trauma, 5 with Lyme arthritis, and 3 with rheumatoid arthritis, and passaged at least 6 times prior to stimulation. Cells were stimulated with 10 µg/ml of *S. aureus* PG muramyl dipeptide (Sigma-Aldrich) or media alone (ctrl) for 24 h. Shown are mean (+/- SD) and estimation plots of pro-inflammatory (IL-6, IL-8, IL-1β, TNF⍺, IL-12 p70) and anti-inflammatory/pro-fibrotic (IL-10, IL-4, TGF-β1) cytokines detected in cell culture supernatant by multiplex assay. Statistically significant differences between control and PG-stimulated cells were determined by paired two-tailed t test (p values and mean of differences are indicated in figure). Results stratified by disease type are available in supplemental material

Synovial fibroblasts are the major cell type within the joint synovium. To determine the inflammatory responses of these tissue-resident cells, we isolated primary human synovial fibroblasts from 8 patients with osteoarthritis, collected as part of this study, as well as 5 with Lyme arthritis (LA) and 3 with rheumatoid arthritis (RA), collected previously. Cells were incubated in low-serum media and stimulated with the PG NOD2 ligand muramyl dipeptide from *S. aureus* for 24 h. Cell supernatants were collected, and cytokines were analyzed by multiplex assay. PG-stimulated cells secreted elevated levels of numerous cytokines associated with inflammation (IL-1β, TNFα, IL-6, IL-8, IL-12p70) and tissue repair and fibrosis (IL-10, IL-4, TGF-β1). Of these cytokines, IL-6 levels were most significantly altered (*p* < 0.0001), with a ∼ 4-fold increase in supernatants from PG-stimulated cells, compared with media alone controls (Fig. 5). These results were consistent with our ex vivo data (Fig. 3). Interestingly, results were similar across different types of synovial fibroblasts (OA vs. LA vs. RA, supplemental Fig. S2). We were able to obtain primary fibroblasts from two patients with joint trauma, and stimulation with PG failed to induce a statistically significant cytokine response, suggesting differences in immune responses between “healthy” and diseased cells, but this observation is subject to over-interpretation and should be treated with caution due to the small number of non-arthritis samples available.

## Discussion

This study provides evidence that peptidoglycan (PG), a bacterial cell wall component, is present in the synovial tissue of over half of patients undergoing primary total knee arthroplasty for degenerative osteoarthritis. Furthermore, our results suggest that PG may play a role in the symptomatology and disease progression of osteoarthritis, as levels of PG positively correlated with synovitis and pro-inflammatory cytokine levels as well as younger age at time of arthroplasty. These results indicate that PG, likely derived from the microbiome, is involved in pathogenesis of synovial inflammation in advanced knee OA for at least a subset of patients undergoing TKA. PG is a pathogen-associated molecular pattern (PAMP) that is recognized by several immune receptors that yield a pro-inflammatory response [18, 20, 21]. Synovitis has been linked to clinical progression of OA [12, 13, 27, 28].

Historical paradigms held that the joint space was free of microbes and microbial debris in the absence of clinical infection, yet data has suggested that immune responses mediated by microbial byproducts may play a role in arthritis. The concept of microbial debris as a mediator of joint inflammation first emerged regarding inflammatory arthritis [24, 29–32]. Newer data indicates that microbial debris, including PG as well as bacterial DNA fragments, is present in joint tissue in degenerative arthritis [17, 22, 23, 33]. Supporting evidence for the role of microbial debris as a mediator of synovitis includes a study showing positive correlation between the PAMP lipopolysaccharide and knee OA severity [34]. PG in particular has been shown in animal models to be strongly arthritogenic [17, 35, 36] and may be exploited as a potential therapeutic target [37]. Our study is the first of this size to quantify PG in a cohort of patients with advanced knee OA and to characterize PG’s association with synovitis and inflammation. Together, our data and previous studies strongly support the premise that microbial debris derived from the host microbiome can act as a driver of synovitis in knee osteoarthritis.

There are several plausible mechanisms by which bacteria or bacterial byproducts from the host microbiome could travel to the knee joint hematogenously. PG has been identified in the blood of healthy individuals without clinical infection [38, 39]. Potential sources of PG include gastrointestinal (GI), oral, and skin flora. Translocation of bacteria from the gastrointestinal tract through a permeable gut barrier has been postulated as a driver of surgical site infections [40]. Gut microbiota, intestinal permeability, GI inflammation, and other gut-associated factors are proposed to contribute to OA, so this phenomenon could also occur in the absence of clinical infection and could include bacterial byproducts [16, 41, 42]. Boer et al. found that gut dysbiosis is associated with joint pain and inflammation [43]. Obesity, known to be strongly associated with OA, is linked to alterations in the gut microbiome that promotes increased absorption of bacterial byproducts [44, 45], although further studies are required to identify the source of PG in synovial tissue. Bacteria or bacterial byproducts may travel directly through the gut barrier or could travel inside white blood cells [46, 47]. An OA microbiome from Goswami et al. suggested that the skin microbiome may also contribute to contamination of microbes within arthritic joints of some patients [48]. Moentadj et al. described the ability of PG-polysaccharide polymers from oral streptococcal species to induce arthritis in mice [35].

We found PG staining of synovium colocalized with both macrophages and synovial fibroblasts. In seeming contradiction, Schrijver et al. [24]. previously showed localization of PG staining from synovial samples only within cells expressing markers of antigen presentation (HLA-DR, CD40, CD80, CD86) *in situ.* However, we and others have subsequently shown that synovia from Lyme arthritis [17] and rheumatoid arthritis [49] contain distinct populations of HLA-DR + CD90 + synovial fibroblasts, particularly within the synovial sub-lining and perivascular regions. The CD90 + cells with fibroblast morphology containing bacterial PG in this study display phenotypically similar characteristics. Our in vitro results further demonstrate that PG induces an inflammatory and fibrotic response in synovial fibroblasts, similar to senescent fibroblasts in other chronic inflammatory and fibrotic diseases [50]. This is further supported by previous *ex-vivo* findings in PG-infected synovial cells [24]. These data support a dual role for synovial fibroblasts, and likely other tissue-resident immune cells, as mediators of the pathogenic response to PG in synovium via upregulation of pro-inflammatory and pro-fibrotic cytokines.

We found no associations between PG and patient reported outcome measures following surgery. While the possibility of type II error cannot be excluded, we do not detect a strong signal that PG present at time of surgery is prohibitive of good outcome following knee replacement surgery. Nonetheless, further investigation of a possible role for PG to adversely affect post-TKA outcomes is warranted. There were no occurrences of periprosthetic joint infection in our elective TKA cohort out to 1 year following surgery. This indicates that the PG identified at time of surgery was not indicative of active clinical infection, but instead represented prior intrusion of these PAMPs into the joint space.

There are several limitations with this study. First, the use of polyclonal antisera limited us to identification of PG antigens restricted to the immunogens used to generate the mouse antisera. Although we selected bacterial species with different PG structures for this study, it is possible that there exists PG in the ‘negative’ patients that may be detected by more sensitive techniques. Furthermore, this study only examined a small portion of discarded synovial tissue from each patient, and there are likely other PG ‘negative’ patients that may have PG in other regions of the synovium. Another limitation is that the assay used was unable to distinguish between different types of PG in patients’ synovia, and it is unknown whether we were detecting PG from intact bacteria, or PG fragments alone. Other studies have found a distinct shift in microbial DNA from gram-positive bacteria to gram-negative bacteria in OA and distinct microbial signatures based on hospital of origin and prior intraarticular steroid injection [23, 48]. The last observation also indicates that skin microbiota may be contributing to PG contamination into the joint environment. Furthermore, the type of.

PG may have a marked impact on cellular phenotypes of nearby cells within the synovial microenvironment. Spatial imaging approaches, analysis of microbial DNA, and use of PG antibodies specific for distinct PG types may be needed to resolve some of these outstanding questions. Another limitation is the types of primary fibroblasts available for this study. We were not able to obtain synovial tissue from healthy patients, which would be an ideal control. We were able to obtain primary fibroblasts from two patients with knee trauma who underwent TKA. Differences in cytokine levels between stimulated vs. unstimulated cells from trauma patients did not achieve statistical significance (Supplemental Figure S2), but interpretation of these data are difficult because of the low sample number in this subgroup. Further experiments using more sensitive techniques and reagents will be needed to resolve these outstanding questions.

## Conclusions

In this study, we identified bacterial PG in patient synovium from over half (33/56) of patients with advanced knee OA undergoing arthroplasty. PG-positive cells included both synovial macrophages and synovial fibroblasts. Furthermore, PG levels positively correlated with inflammatory markers, including inflammatory synovitis severity and elevated levels of IL-6 in synovial fluid. This ex vivo observation was supported by in vitro stimulation of primary human synovial fibroblasts with PG, which secreted high levels of both pro-inflammatory and pro-fibrotic cytokines, most notably IL-6. These findings implicate bacterial PG as an important contributor of joint inflammation and synovial tissue damage in OA. Further research is warranted to explore PG as a potential diagnostic and/or therapeutic target.

### Electronic supplementary material

Below is the link to the electronic supplementary material.

Supplemental figure S1: Bacterial peptidoglycan in synovial tissue is localized within both mononuclear and fibroblastic cells. PG-immunostaining of (A) primary TKA synovial tissue or (B) synovial tissue with S. aureus infection are shown. Arrows indicate examples of PG-positive cells with a mononuclear morphology, and triangles indicate examples of PG-positive cells with a fibroblastic morphology. Numbers in panels on right correspond to arrows and triangles in figure.

Supplemental figure S2: Cytokine secretion by primary human synovial fibroblasts stimulated with peptidoglycan (PG), stratified by primary diagnosis. Primary human synovial fibroblasts were isolated from 4 patients with Lyme arthritis (LA), 3 with rheumatoid arthritis (RA), 8 patients with osteoarthritis (OA), 2 with joint trauma (OA and trauma combined in ?Other), passaged at least 6 times prior to stimulation. Cells were stimulated with 10 ?g/ml of S. aureus PG muramyl dipeptide (Sigma-Aldrich) or media alone (ctrl) for 24 hours. Shown are mean (+/- SD) of pro-inflammatory and anti-inflammatory/pro-fibrotic cytokines detected in cell culture supernatant by multiplex assay. Statistically significant differences between control and PG-stimulated cells were determined by paired two-tail t test (p values are indicated in figure).

## Data Availability

All data generated or analyzed during this study are included in this published article and its supplementary information files.

## Abbreviations

H&E: Hematoxylin and eosin
IHC: Immunohistochemistry
IFM: Immunofluorescence microscopy
KOOS JR: Knee injury and Osteoarthritis Outcome Score for Joint Replacement
LA: Lyme arthritis
OA: Osteoarthritis
PAMP: Pathogen-associated molecular pattern
PG: Peptidoglycan
RA: Rheumatoid arthritis
TKA: Total knee arthroplasty
VR-12: Veterans Rand-12 Health Survey
