# Supplementary figures and images for: Peptidoglycan in osteoarthritis synovial tissue is associated with joint inflammation
Source: Arthritis Res Ther. 2024 Mar 27;26:77. doi: 10.1186/s13075-024-03293-x (PMC10967045; doi:10.1186/s13075-024-03293-x)

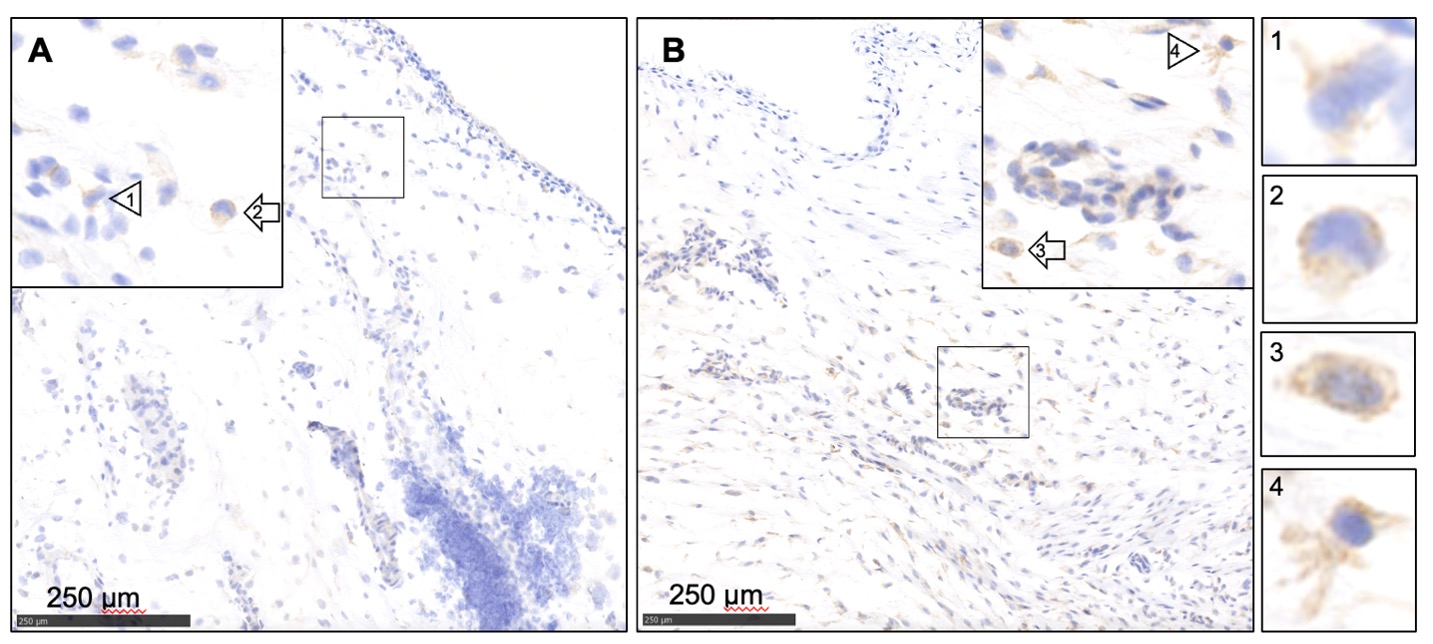

Supplement: Supplementary file 1 — Supplemental figure S1: Bacterial peptidoglycan in synovial tissue is localized within both mononuclear and fibroblastic cells. PG-immunostaining of (A) primary TKA synovial tissue or (B) synovial tissue with S. aureus infection are shown. Arrows indicate examples of PG-positive cells with a mononuclear morphology, and triangles indicate examples of PG-positive cells with a fibroblastic morphology. Numbers in panels on right correspond to arrows and triangles in figure. [file 13075_2024_3293_MOESM1_ESM.jpg]

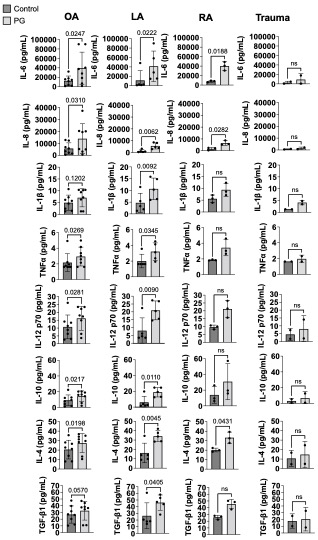

Supplement: Supplementary file 2 — Supplemental figure S2: Cytokine secretion by primary human synovial fibroblasts stimulated with peptidoglycan (PG), stratified by primary diagnosis. Primary human synovial fibroblasts were isolated from 4 patients with Lyme arthritis (LA), 3 with rheumatoid arthritis (RA), 8 patients with osteoarthritis (OA), 2 with joint trauma (OA and trauma combined in ?Other), passaged at least 6 times prior to stimulation. Cells were stimulated with 10 ?g/ml of S. aureus PG muramyl dipeptide (Sigma-Aldrich) or media alone (ctrl) for 24 hours. Shown are mean (+/- SD) of pro-inflammatory and anti-inflammatory/pro-fibrotic cytokines detected in cell culture supernatant by multiplex assay. Statistically significant differences between control and PG-stimulated cells were determined by paired two-tail t test (p values are indicated in figure). [file 13075_2024_3293_MOESM2_ESM.jpg]
